# Pterygium and genetic polymorphisms of the DNA repair enzymes XRCC1, XPA, and XPD

**Published:** 2010-04-20

**Authors:** Chun-Chi Chiang, Yi-Yu Tsai, Da-Tian Bau, Ya-Wen Cheng, Sung-Huei Tseng, Rou-Fen Wang, Fuu-Jen Tsai

**Affiliations:** 1 Department of Ophthalmology, China Medical University Hospital, Taichung, Taiwan; 2 Institute of Medical Molecular Toxicology, Chung Shan Medical University, Taichung, Taiwan; 3 Terry Fox Cancer Research Laboratory, China Medical University Hospital, Taichung, Taiwan; 4 Department of Medical Genetics, China Medical University Hospital, Taichung, Taiwan; 5 Institute of Medicine, Chung Shan Medical University, Taichung, Taiwan; 6 Department of Ophthalmology, National Cheng Kung University Hospital, Tainan, Taiwan; 7 Department of Biotechnology and Bioinformatics, Asia University, Taichung, Taiwan

## Abstract

**Purpose:**

Pterygium is an ultraviolet (UV) related disease. UV radiation can produce DNA damage, which is repaired by the DNA repair systems. Among the DNA repair systems, the base excision repair (BER) and nucleotide excision repair (NER) systems are the major ones involved in repairing UV-induced DNA damage; X-ray repair cross complementary 1 (*XRCC1*) and human 8-oxoguanine DNA glycosylase 1 (h*OGG1*) are two BER genes, and xeroderma pigmentosum group A (*XPA*) and xeroderma pigmentosum group D (*XPD*) are two NER genes. Polymorphisms of these genes are associated with the differences in their repair DNA damage capacity, and they modulate the susceptibility to cancer. Because the polymorphism of h*OGG1* was reported to be associated with pterygium, it is logical to assume the correlation between *XRCC1*, *XPA*, and *XPD* polymorphisms and pterygium formation.

**Methods:**

One hundred and twenty-seven pterygium patients and 103 volunteers without pterygium were enrolled in this study. Polymerase chain reaction based analysis was used to resolve the *XRCC1* codon 107, 194, 280, and 399; *XPA* A23G; *XPA* codon 228; and *XPD* codon 751 polymorphisms.

**Results:**

There were significant differences in the frequency of genotypes and alleles of *XRCC1* codon 194 and 399 polymorphisms between the groups. In codon 194, individuals who carried at least 1 Trp allele had a decreased risk of developing pterygium compared to those who carried the Arg/Arg wild-type genotype (odds ratio [OR]=0.58; 95% CI: 0.34–0.98). In codon 399, individuals who carried at least 1 Gln allele had a threefold increased risk of developing pterygium compared to those who carried the Arg/Arg wild-type genotype (OR=3.06; 95% CI: 1.78–5.26). There were no significant differences in the frequency of the genotypes and alleles of *XRCC1* codon 107 and 280, *XPA* A23G, and *XPD* codon 751 polymorphisms between the groups. The *XPA* codon 228 polymorphism was not detected in any of the cases or controls.

**Conclusion:**

The *XRCC1* codon 194 polymorphism causes a decreased risk of developing pterygium, but the codon 399 polymorphism increases the risk. There is no correlation between pterygium and *XRCC1* codon 107 and 280, *XPA* A23G, and *XPD* codon 751 polymorphisms.

## Introduction

Although the pathogenesis of pterygia is still under investigation, epidemiological evidence suggests that ultraviolet (UV) irradiation plays the most important role [1-3]. Moreover, after abnormal levels of the tumor suppression protein, p53 protein and *p53* gene mutation were found in the epithelium, an increasing number of researchers felt that pterygium is a UV-related, uncontrolled cell proliferation consistent with that of a tumor [4-8].

UV irradiation can produce DNA damage, which will lead to gene mutation and uncontrolled cell proliferation [9-11]. Most DNA damage is repaired by the DNA repair system. In humans, more than 70 genes are involved in five major DNA repair pathways: direct repair, base excision repair (BER), nucleotide excision repair (NER), mismatch repair, and double strand break repair [12,13]. The NER and BER systems are the major repair systems involved in repairing UV-related DNA damage [11].

X-ray repair cross complementary 1 (*XRCC1*) is a major gene in the BER system, and xeroderma pigmentosum group A (*XPA*) and xeroderma pigmentosum group D (*XPD*) are two important genes in NER system [11,14,15]. Polymorphisms of the genes have an effect on the host’s capacity to remove DNA damage and are reported to modulate the susceptibility to cancer [16-21].

Because there is evidence that genetic factors and UV-induced damage play a role in the development of pterygium [1,22,23]—genetic predisposition to pterygium was recently discovered using the single nucleotide polymorphism (SNP) method—and the polymorphisms of DNA repair genes have an effect on host capacity of removing DNA damage, it is logical to assume the correlation between pterygium and the genetic polymorphisms of DNA repair enzymes XRCC1, XPA, and XPD.

In this study, we selected polymorphisms in the 3 genes based upon conservation of the gene sequence through evolution, frequency, and those occurring in exons resulting in amino acid changes of potential functional significance [24-26]. There were four polymorphism sites studied in *XRCC1*: codon 107, 194, 280, and 399. Among them, most of the published codon 194 polymorphism—an arginine (Arg) to Tryptophan (Trp) change (C→T, Arg194Trp)—studies reported a reduced risk of cancer associated with the T allele [25]. Codon 399 polymorphism—an arginine (Arg) to glutamine (Gln) change (G→A, Arg399Gln)—was never reported to be associated with cancer formation and deficient DNA repair [19,25,27,28]. Only relatively small studies have assessed that the less common codon 280 polymorphism and its association with cancer was controversial [25]. There are no reports about the codon 107 polymorphism and the risk of tumors. There were two polymorphism sites identified in *XPA*: A23G and codon 228 (G709A, Arg228Gln) [29]. Several polymorphisms were reported in *XPD*, but *XPD* codon 751 polymorphism (A→C, Lys751Gln) was reported to affect the proficiency of DNA repair [18,20].

In the present study, we conducted a case control study to evaluate the associations of pterygium formation and *XRCC1* codon 107, 194, 280, and 399; *XPA* A23G; *XPA* codon 228; and *XPD* codon 751 polymorphisms.

## Methods

### Patients

A total of 127 pterygium patients (70 males and 57 females) at the Department of Ophthalmology, National Cheng- Kung University Hospital (Tainan, Taiwan) from January 2003 to June 2003 were enrolled in the study, with ages ranging from 35 to 90 years (mean: 64.6 years). Patients included in this study were apex of pterygium invading the cornea for more than 1 mm. One hundred and three volunteers aged 50 years or more without pterygium were enrolled as the control group. There were 64 males and 39 females in the control group (age range of 50 to 83 years with an average age of 64.2). This study was performed with approval from the Human Study Committee of the China Medical University Hospital and National Cheng Kung University Hospital. Informed consent was obtained from all individuals who participated in this study.

Genomic DNA was prepared from peripheral blood by use of a DNA Extractor WB kit (Wako, Japan). Polymerase chain reactions (PCRs) were performed in a total volume of 25 µl, containing genomic DNA; 2–6 pmol of each primer; 1× Taq polymerase buffer (1.5 mM MgCl_2_); and 0.25 units of AmpliTaq DNA polymerase (Perkin Elmer, Foster City, CA).

### *XRCC1* codon 107, 194, 280, and 399 polymorphisms

The PCR conditions for *XRCC1* codon 107, 194, 280, and 399 polymorphisms were designed by us using EntrezGene. The PCR condition was initiated by a 5 min denaturation step at 95 °C, followed by 35 cycles at 95 °C for 30 s, 58 °C, 58 °C, 63 °C, 60 °C for *XRCC1* codon 107, 194, 280, and 399, respectively, for 30 s, 72 °C for 30 s, and a final step at 72 °C for 7 min. The PCR products were subjected to restriction digestion overnight at 37 °C by RsaI, HpaII, NciI, and MspI for *XRCC1* codon 107, 194, 280, and 399, respectively. The primers and probes for *XRCC1* polymorphisms are listed in Table 1.

**Table 1:** The primers and probes for *XRCC1*, *XPA*, and *XPD* polymorphisms.

**Polymorphism**	**Primer sequence**
*XRCC1* codon 107	F: 5'-TTGACCCCCAGTGGTGCT-3’R: 5'-AGTCTGCTGGCTCTGGGCTGG-3'
*XRCC1* codon 194	F: 5'-CAGACAAAGATGAGGCAGAGG-3'R: 5'-TCAGACCCAGGAATCTGAGC-3'
*XRCC1* 280	F: 5'-CATCTCTCCCTTGGTCTCCA-3’R: 5'-CAGGATAAGGAGCAGGGTTG-3'
*XRCC1* 399	F: 5'-GGACTGTCACCGCATGCGTCGG-3'R: 5'-GGCTGGGACCACCTGTGTT-3'
*XPA* A23G	F: 5’-TTAACTGCGCAGGCGCTCTCACTC-3’R: 5’-AAAGCCCCGTCGGCCGCCGCCAT-3’
*XPA* codon 228	F: 5’-TTTTCAGAATTGCGTC-3’R: 5’-TTCATATGTCAGTTCATG-3’
*XPD* codon 751	F: 5’-GCCCGCTCTGGATTATACG-3’R: 5’-CTATCATCTCCTGGCCCCC-3’

In the table, "F" indicates forward primer and "R" indicates reverse primer.

### *XPA* A23G, *XPA* codon 228, and *XPD* codon 751 polymorphisms

The method of determining the *XPA* A23G, *XPA* codon 228, and *XPD* codon 751 genotypes was the same as the previous study [17,20]. The PCR primers for the *XPA* A23G, codon 228 and *XPD* codon 751 polymorphism are list in Table 1. Briefly, PCR reactions were performed in a 20-μl reaction volume containing 200 ng of genomic DNA, 10 pmol of each primer, 0.2 mm  each deoxynucleotide triphosphate, 1× PCR buffer (75 mm  Tris-HCl [pH 9.0], 15 mm ammonium sulfate, and 0.1 μg/μl BSA), 2.5 mm MgCl_2_, and 1 unit of Taq polymerase (Takara Shuzo Co., Otsu, Shiga, Japan). The mixture were amplified with a Perkin-Elmer GeneAmp PCR System 9600 (Perkin-Elmer, Foster, CA). The PCR profile consisted of an initial melting step of 94 °C for 5 min, followed by 36 cycles of denaturation at 94 °C for 20 s; primer annealing, 20 s at 58 °C for A23G and 20 s at 48 °C for codon 228; and primer extension, 20 s at 72 °C for A23G and 30 s at 72 °C for codon 228. The cycles were followed by a final elongation step at 72 °C for 5 min for A23G and 10 min for codon 228. The PCR products were checked on a 2% agarose gel, photographed using Polaroid film, and were then subjected to RFLP analysis.

The PCR products from the same individual were mixed together and 10 µl of this solution was loaded into 3% agarose gel containing ethidium bromide for electrophoresis.

Statistical analysis for the distributions of the *XRCC1* codon 107, 194, 280, 399, *XPA* A23G, *XPA* codon 228, and *XPD* codon 751 polymorphisms in the control group and pterygium group were compared using the χ^2^ test. The risk of pterygium was estimated using the odds ratio (OR) and a 95% confidence interval (CI).

## Results

There were no significant differences between the groups in age or sex. The frequencies of the genotypes and alleles of *XRCC1* codon 107, 194, 280, and 399 polymorphisms in the pterygium group and control group are shown in Table 2 and Table 3. There were significant differences between the groups in codon 194 and 399 polymorphisms and no differences in codon 107 and 280.

**Table 2:** Genotypes and allelic frequencies for *XRCC1* codon 399 polymorphism (G→A, Arg399Gln) in the pterygium and control group.

**Polymorphism**	**Pterygium total=127 (%)**	**Control total=103 (%)**	**OR (95% CI)**
Genotype			
GG	48 (37.8)	67 (65.0)	1
GA	70 (55.1)	31 (30.1)	3.152 (1.796–5.531)
AA	9 (7.1)	5 (4.9)	2.513 (0.792–7.969)
GA or AA	79 (62.2)	36 (35.0)	3.063 (1.783–5.262)
Allele			
G	166 (65.4)	165 (80.1)	1
A	88 (34.6)	41 (19.9)	2.133 (1.390–3.275)

**Table 3:** Distribution of allelic frequency of *XRCC1* codon 107, 194, 280, 399, XPA A23G and XPD codon 751 polymorphisms.

**Polymorphism**	**Pterygium 127**	**Control 103**	**p**	**OR**
XRCC1 codon 107 (A→G)			0.82	
Allele A	203 (79.9%)	162 (78.6%)		1
Allele G	51 (20.1%)	44 (21.4%)		0.925 (0.589–1.452)
XRCC1 codon 194 (C→T)			0.0001	
Allele C	167 (65.8%)	98 (47.6%)		1
Allele T	87 (34.2%)	108 (52.4%)		0.473 (0.325–0.689)
XRCC1 codon 280 (G→A)			0.51	
Allele G	189 (74.4%)	159 (77.2%)		1
Allele A	65 (25.6%)	47 (22.8%)		1.163 (0.758–1.787)
XRCC1 codon 399 (G→A)			0.001	
Allele G	168 (66.1%)	165 (80.1%)		1
Allele A	86 (33.9%)	41 (19.9%)		2.060 (1.343–3.160)
XPA A23G (A→G)			0.54	
Allele A	122 (48%)	93 (45.1%)		1
Allele G	132 (52%)	113 (54.9%)		0.89 (0.616–1.287)
XPD codon 751 (A→C)			0.17	
Allele A	235 (92.5%)	197 (95.6%)		1
Allele C	19 (7.5%)	9 (4.4%)		1.77 (0.797–3.924)

In the *XRCC1* codon 194 polymorphism (C→T, Arg194Trp), the odds ratio of C/T polymorphism was 0.979 and T/T polymorphism was 0.292 compared to the C/C wild-type genotype. Hence, individuals who carried at least 1 T (Trp) allele (C/T and T/T) had a decreased risk, or a protective effect, of developing pterygium compared to those who carried the C/C (Arg/Arg) wild-type genotype (OR=0.58; 95% CI: 0.34–0.98). Those who carried allele T had a decreased risk, or a protective effect, of developing pterygium compared to those who carried allele C (OR=0.473; 95% CI: 0.325–0.689).

In the *XRCC1* codon 399 polymorphism (G→A, Arg399Gln), the odds ratio of G/A polymorphism was 3.15 and A/A polymorphism was 2.51 compared to the G/G wild-type genotype. Hence, individuals who carried at least 1 A (Gln) allele (G/A and A/A) had a threefold increased risk of developing pterygium compared to those who carried the G/G (Arg/Arg) wild-type genotype (OR=3.06; 95% CI: 1.78–5.26). Those who carried allele A had a twofold increased risk of developing pterygium compared to those who carried allele G (OR=2.13; 95% CI: 1.39–3.28).

The frequency of the genotypes and alleles of *XPA* A23G and *XPD* codon 751 polymorphisms in the pterygium group and control group is shown in Table 2 and Table 3. There were no significant differences between the groups. The *XPA* codon 228 (G709A, Arg228Gln) polymorphism was not detected in the cases or controls; all patients were GG (Arg/Arg) wild-type genotypes.

## Discussion

This is the first case-control study of polymorphisms in *XRCC1* codon 107, 194, 280, and 399; *XPA* A23G; *XPA* codon 228; and *XPD* codon 751 in relation to pterygium formation. In our study, we found that the *XRCC1* codon 194 polymorphism was associated with a decreased risk of developing pterygium, and that the codon 399 polymorphism was associated with susceptibility to pterygium. The *XRCC1* codon 107 and 280, *XPA* A23G, and *XPD* codon 751 polymorphisms were not associated with pterygium formation. The *XPA* codon 228 (G709A) polymorphism was not detected in the cases and controls; we suggest that this is due to ethnic differences, as in the report of Park et al. [17].

*XRCC1*, *XPA*, and *XPD* are all DNA repair genes. The difference between *XPA* and *XPD*, and *XRCC1* is that *XPA* and *XPD* are NER, and *XRCC1* is BER. The BER pathway repairs small base adducts that are produced by oxidation, methylation, and radiation, whereas the NER pathway repairs bulky and helix-distorting adducts induced by chemical carcinogens and radiation [11-13]. The noxious effects of UV irradiation on DNA are either directly by the UV phototoxic effect or indirectly by oxidative stress [9-11]. Both the UV phototoxic effect and oxidative stress mainly produce single base changes and tandem mutations in DNA [11,30]. Single base changes are normally reversed by the BER system and tandem mutations are repaired by the NER system [11,30-33]. Hence, among the five major DNA repair systems, NER and BER are the main systems involved in repairing UV-induced DNA damage [11].

Because pterygium is a UV-induced disease, we assume that both BER and NER play a role in pterygium formation. *XRCC1* and human 8-oxoguanine DNA glycosylase 1 (h*OGG1*) are BER system genes and *XRCC1*, in our series, and h*OGG1*, in the report of Kau et al. [22] , are found to be associated with pterygium formation; therefore, the BER system indeed plays an important role in pterygium formation. However, *XPA* and *XPD*— NER system genes—are not found to be associated with pterygium in our series. We suggest that the different result may be due to the following three possibilities: First, it is other polymorphisms in *XPA* and *XPD* and not the *XPA* A23G, *XPA* codon 228, and *XPD* codon 751 polymorphisms that are involved in pterygium formation. Second, it is other genes in NER, not *XPA* or *XPD*, that are involved in pterygium formation. Third, it is BER and other DNA repair systems, not NER, that play a role in pterygium formation.

NER is a complex process involving more than 30 gene products, and *XPA* and *XPD* are two important genes among these [11]. *XPA* plays a central role in NER through its interaction with the replication protein A, transcription factor TFIIH, and excision repair cross-complementing group 1-XPF [11,14,15]. *XPD* encodes a helicase, which participates in both NER and basal transcription as part of the transcription factor TFIIH [33]. Polymorphisms of *XPA* A23G and *XPD* codon 751 were reported to have a modulating effect on NER capacity [16,18,20]. If the *XPA* A23G and *XPD* codon 751 polymorphisms, like in other tissues, are correlated with the DNA repair capacity of NER in the conjunctiva and cornea, the result of polymorphisms of *XPA* A23G and *XPD* codon 751 not associated being with pterygium formation may suggest that the NER system might not play a role in pterygium formation; however, more evidence is required to support this theory.

Analysis of the gene mutation spectrum in pterygium is a method of evaluating the role of BER and NER in pterygium. We suggest that UV radiation can produce single base changes and tandem mutations in the conjunctiva and cornea, as in other tissues. When both NER and BER are normal in the conjunctiva and cornea, all mutations can be repaired. However, when the NER system is intact, but the BER system has reduced activity, only tandem mutations are repaired and single base changes are not, and vice versa. In our previous study, all *p53* gene mutations in pterygium were single base changes, not tandem mutations [4]. Hence, we suggest that the function of the NER system is normal in pterygium, but that of the BER system has reduced activity. If the NER system in pterygium is the same as that of normal people, it is reasonable that *XPA* and *XPD* polymorphisms were not found to be differently distributed between those with pterygium and the normal controls. If pterygium is associated with reduced activity of the BER system, it is reasonable that the *XRCC1* and h*OGG1* polymorphisms were found to be differently distributed between those with pterygium and the normal controls. This is because the polymorphisms of *XRCC1* and h*OGG1* have a different DNA repair capacity [19,28], and people who have polymorphism with lower DNA repair activity are prone to pterygium.

Among *XRCC1* polymorphisms, codon 399 polymorphism was well studied and was found to be associated with formation of several cancers [19,27,34,35], but the exact biochemical effect of the polymorphism is not fully characterized. *XRCC1* plays a pivotal role in BER by bringing together DNA polymerase-β, DNA ligase III, and PARP at the site of DNA damage. The codon 399 variant lies within the BRCT-1 domain of XRCC1. The BRCT-1 domain is a region with extensive homology to BRCA1 and includes a binding site for PARP [12,13,36]. Therefore, the amino acid substitutions in codon 399 are proposed to change the function of the protein, and the 399Gln allele of *XRCC1* has an important and potentially harmful phenotype and is reported to be associated with cancer [19,27,28,34-37]. Our finding of a positive association between the *XRCC1* codon 399Gln allele and pterygium is consistent with the published, functional studies [28,37].

Sturgis et al. [34] reported an OR of 1.6 (95% CI, 1.0–2.6) for the *XRCC1* codon 399 Gln/Gln genotype in a case-control study of head and neck cancer, and Divine et al. [35] observed an odds ratio of 2.8 (95% CI, 1.2–7.9) for *XRCC1* codon 399 Gln/Gln genotype in a case-control study of lung cancer. However, there was study that reported contrary findings: Stern et al. [27] observed an inverse association between *XRCC1* codon 399 Gln/Gln genotype and bladder cancer. In our series, individuals who carried at least 1 A (Gln) allele had a threefold increased risk (OR=3.06; 95% CI: 1.78–5.26) of developing pterygium compared to those who carried the GG (Arg/Arg) wild-type genotype, which is consistent with most epidemiological studies [19,28,34,35,37].

A second *XRCC1* polymorphism (Arg194Trp) has also been well studied, and most of the published codon 194 polymorphism studies reported a reduced risk of cancer associated with the Trp allele [25,27,34,38]. The reported odd ratio was from 0.4 to 0.7 [25,27,34,38]. In our series, individuals who carried at least 1 T (Trp) allele (C/T and T/T) also had a decreased risk of developing pterygium compared to those who carried the C/C (Arg/Arg) wild-type genotype (OR=0.58; 95% CI: 0.34–0.98), which is consistent with most of the published studies reporting inverse associations between the *XRCC1* 194Trp allele and cancer risk at many sites [25].

*XRCC1* codon 194 polymorphism results from a transition from a positively charged Arg to a hydrophobic Trp, which may alter XRCC1 function; however, human studies of XRCC1 Arg194Trp have reported no associations with the indicators of DNA-repair capacity, such as DNA-adduct levels, frequency of mutations in glycophorin A, or sensitivity to ionizing radiation [28,39,40]. Hence, the mechanism of this polymorphism’s inverse association with cancer and pterygium is still unknown. Because the functional significance of the Arg194Trp region is not clear, more studies are needed to define its role.

There were two limitations in our study. First, we didn’t stratify the study population in terms of severity. There were numerous classification systems in pterygium, e.g., atrophic, intermediate, and fleshy type based on the relative translucency of the body. While pooling all of the patients’ data together provided a general overview of the risks of genetic polymorphism, we suggest that the dichotomy of our patients’ data according to classification systems might provide further insight into the pathogenesis of pterygium. Further study is recommended. Second, we could not show that the AA polymorphism of *XRCC1* codon 399 created a significant risk of developing pterygium (OR=2.513; 95% CI: 0.792–7.969). Because there are only nine pterygium patients and five normal controls with AA polymorphism, we can’t have the significantly different result in analysis of this genotype due to the small number of the sample. Hence, we could not show that AA polymorphism created a significant risk of developing pterygium, and we also could not show that the presence of GA polymorphism partially negates the risks of developing pterygium when compared with AA. However, we found that the presence of allele A meant a twofold increased risk of developing pterygium compared to allele G, and individuals who carried at least 1 A (Gln) allele (GA and AA) had a threefold increased risk of developing pterygium compared to those who carried the GG (Arg/Arg) wild-type genotype. These results support the theory that the *XRCC1* codon 399 polymorphism is associated with pterygium.

In conclusion, our study demonstrated for the first time that the *XRCC1* codon 194 polymorphism is inversely associated with pterygium, and that the codon 399 polymorphism is associated with the risk of pterygium. Polymorphisms of *XPA* A23G and *XPD* codon 751 are not associated with pterygium formation. Further studies on the polymorphisms of other genes in NER, BER, or other DNA repair systems are necessary for the detection of a genetic predisposition to pterygium formation and for the investigation of the roles of NER, BER, or other DNA repair genes in pterygium formation.
